# Author Correction: Saliva is more sensitive than nasopharyngeal or nasal swabs for diagnosis of asymptomatic and mild COVID-19 infection

**DOI:** 10.1038/s41598-021-89880-3

**Published:** 2021-06-09

**Authors:** Alvin Kuo Jing Teo, Yukti Choudhury, Iain Beehuat Tan, Chae Yin Cher, Shi Hao Chew, Zi Yi Wan, Lionel Tim Ee Cheng, Lynette Lin Ean Oon, Min Han Tan, Kian Sing Chan, Li Yang Hsu

**Affiliations:** 1 Saw Swee Hock School of Public Health, National University of Singapore, National University Health System, #10-01, 12 Science Drive 2, Singapore, 117549 Singapore; 2 Lucence Diagnostics, Singapore, Singapore; 3 Department of Medical Oncology, National Cancer Centre, Singapore, Singapore; 4 Duke-NUS Graduate Medical School, National University of Singapore, Singapore, Singapore; 5 Genome Institute of Singapore, Singapore, Singapore; 6 Headquarters Army Medical Services, Singapore Armed Forces, Singapore, Singapore; 7 Department of Diagnostic Radiology, Singapore General Hospital, Singapore, Singapore; 8 Department of Molecular Pathology, Singapore General Hospital, Singapore, Singapore; 9 Yong Loo Lin School of Medicine, National University of Singapore, National University Health System, Singapore, Singapore

Correction to: *Scientific Reports* 10.1038/s41598-021-82787-z, published online 04 February 2021

In the original version of this Article, the competing interests statement is incomplete.

The text,

“The authors declare no competing interests.”

Now reads,

“Yukti Choudhury, Chae Yin Cher, Zi Yi Wan, and Min Han Tan are employees of, and affiliated with, Lucence. Y.C. and M.H.T. are shareholders of Lucence; M.H.T. is a director of Lucence. An affiliated entity of Lucence is the applicant of certain patent applications for relevant diagnostics. Y.C., C.Y.C. and/or M.H.T. are named inventors for some of these patent applications. At the time of this research, patents are pending for the next-generation sequencing approach (SG App. No.10202007270P) and the saliva testing process (SG App. No. 10202007826S) discussed in the manuscript.”

This has now been corrected in the PDF and HTML versions of the Article.

